# Supplementary material for: Safe Model-Based Reinforcement Learning for Systems With Parametric Uncertainties
Source: Front Robot AI. 2021 Dec 16;8:733104. doi: 10.3389/frobt.2021.733104 (PMC8717089; doi:10.3389/frobt.2021.733104)
Supplement: Supplementary file 1 [file DataSheet1.pdf]

Supplementary Material

1 CODE TO REPRODUCE SIMULATION RESULTS

Code that reproduces the simulation results can be downloaded from https://github.com/scc-lab/publications-code/tree/master/2021-Frontiers-BT-MBRL.

2 PROOFS OF MAIN RESULTS

LEMMA 1. *If $t \mapsto \Phi\big(t, b(x^0), \zeta\big)$ is a complete Carathéodory solution to* (7)*, starting from the initial condition $b(x^0)$, under the feedback policy $(s,t) \mapsto \zeta(s,t)$ and $t \mapsto \Lambda(t, x^0, \xi)$ is a Carathéodory solution to* (1)*, starting from the initial condition x^0, under the feedback policy $(x,t) \mapsto \xi(x,t)$, defined as $\xi(x,t) = \zeta(b(x), t)$, then $\Lambda(\cdot, x^0, \xi)$ is complete and $\Lambda(t, x^0, \xi) = b^{-1}\left(\Phi(t, b(x^0), \zeta)\right)$ for all $t \in \mathbb{R}_{\geq 0}$.*

PROOF. Note that since $t \mapsto \Phi\big(t, b(x^0), \zeta\big)$ is a complete Carathéodory solution to $\dot{s} = F(s) + G(s)\zeta(s,t)$, it is differentiable at almost all $t \in \mathbb{R}_{\geq 0}$. Since b^{-1} is smooth, $t \mapsto b^{-1}\left(\Phi\left(t, b(x^0), \zeta\right)\right)$ is also differentiable at almost all $t \in \mathbb{R}_{\geq 0}$. That is,

$$\frac{\mathrm{d}(b^{-1} \circ \Phi_i)}{\mathrm{d}t}\left(t, b(x^0), \zeta\right) = \frac{\mathrm{d}b^{-1}_{(a_i,A_i)}(y)}{\mathrm{d}y}\Big|_{y=\Phi_i(t,b(x^0),\zeta)} \frac{\mathrm{d}\Phi_i}{\mathrm{d}t}\left(t, b(x^0), \zeta\right),$$

for almost all $t \in \mathbb{R}_{\geq 0}$ and all $i = 1, \cdots, n$, where Φ_i denotes the ith component of Φ. As a result,

$$\frac{\mathrm{d}(b^{-1} \circ \Phi_i)}{\mathrm{d}t}\left(t, b(x^0), \zeta\right) = \frac{\left(F\left(\Phi\left(t, b(x^0), \zeta\right)\right)\right)_i}{B_i\left(\Phi_i\left(t, b(x^0), \zeta\right)\right)} + \frac{\left(G\left(\Phi\left(t, b(x^0), \zeta\right)\right)\right)_i \zeta\left(\Phi\left(t, b(x^0), \zeta\right), t\right)}{B_i\left(\Phi_i\left(t, b(x^0), \zeta\right)\right)},$$

for almost all $t \in \mathbb{R}_{\geq 0}$ and all $i = 1, \ldots, n$. By the construction of F, G, and ξ,

$$\frac{\mathrm{d}(b^{-1} \circ \Phi)}{\mathrm{d}t}\left(t, b(x^0), \zeta\right) = f\left(b^{-1} \circ \Phi\left(t, b(x^0), \zeta\right)\right)\theta \\ + g\left(b^{-1} \circ \Phi\left(t, b(x^0), \zeta\right)\right)\xi\left(b^{-1} \circ \Phi\left(t, b(x^0), \zeta\right), t\right),$$

for almost all $t \in \mathbb{R}_{\geq 0}$. Clearly $t \mapsto b^{-1} \circ \Phi\left(t, b(x^0), \zeta\right)$ is a Carathéodory solution of (1) on $\mathbb{R}_{\geq 0}$, starting from the initial condition $b^{-1}\big(b(x^0)\big) = x^0$ under the feedback policy $(x,t) \mapsto \xi(x,t)$. By uniqueness of solutions $\dot{x} = f(x)\theta + g(x)\xi(x,t)$ (which follows from local Lipschitz continuity of f, g, and b inside the barrier), $\Lambda(\cdot, x^0, \xi)$ is complete and $\Lambda(t, x^0, \xi) = b^{-1}\left(\Phi\left(t, b(x^0), \zeta\right)\right)$ for all $t \in \mathbb{R}_{\geq 0}$.

LEMMA 2. *If $\|Y_f\|$ is non-decreasing in time then* (13) *admits Carathéodory solutions.*

PROOF. Since $\|Y_f(0)\| = 0$, given any piecewise continuous control signal $t \mapsto u(t)$ and initial conditions s^0 and θ^0, the Cauchy problem $\dot{z} = h_1(z,u)$, $z(0) = z^0 = [s^0; 0; 0; 0; 0; \theta^0]$ admits a unique Carathéodory solution $t \mapsto z_1(0, z^0)$ over $[0, t^*)$, with $t^* = \min(t_1, t_2)$, where $t_1 = \inf\{t \in \mathbb{R}_{\geq 0} \mid \|Y_{f1}(t, z^0)\| = \overline{Y_f}\}$ and $t_2 = \inf\{t \in \mathbb{R}_{\geq 0} | \lim_{\tau \mapsto t} \|z_1(\tau, z^0)\| = \infty\}$, where Y_{f1} denotes the Y_f component of z_1.

Given any $(t', z') \in \mathbb{R}_{\geq 0} \times \mathbb{R}^{2n+2p+p^2+np}$, the Cauchy problem $\dot{z} = h_2(z,u), z(t') = z'$, also admits a unique Carathéodory solution $t \mapsto z_2(t; t', z')$ over $[t', t^{**})$ where $t^{**} = \min\Big(\infty, \big(\inf\{t \in \mathbb{R}_{\geq t'} | \lim_{\tau \mapsto t} \|z_2(\tau, b', z')\| = \infty\}\big)\Big)$.

If $t^* = t_2$ then $t \mapsto z_1(t, z^0)$ is also a unique Carathéodory solution to the Cauchy problem $\dot{z} = h(z,u)$, $z(0) = z^0$. If not, then

$$t \mapsto z^*(t, z^0) = \begin{cases} z_1(t, z^0), & t < t_1, \\ z_2\big(t, t_1, \lim_{\tau \uparrow t_1} z_1(\tau, z^0)\big), & t \geq t_1, \end{cases}$$

is a unique Carathéodory solution to the Cauchy problem $\dot{z} = h(z,u)$, $z(0) = z^0$.

THEOREM 8. *Provided Assumptions 3, 4, and 7 hold, the gains are selected large enough based on* (46) - (49)*, and the weights $\hat{\theta}$, $\hat{W}_c$, Γ, and $\hat{W}_a$ are updated according to* (12), (29), (30)*, and* (31)*, respectively, then the estimation errors $\tilde{W}_c$, $\tilde{W}_a$, and $\tilde{\theta}$ and the trajectories of the transformed system in* (7) *under the controller in* (32) *are locally uniformly ultimately bounded.*

PROOF. Under Assumption 3, the state trajectories are bounded over the interval $[0, T)$. Over the interval $[T, \infty)$, let $B_r \subset \mathbb{R}^{n+2L+p}$ denote a closed ball with radius r centered at the origin. Let χ denote the projection of B_r onto $\mathbb{R}^n$. For any continuous function $h : \mathbb{R}^n \to \mathbb{R}^m$, let the notation $\overline{\|(\cdot)\|}$ be defined as $\overline{\|h\|} := \sup_{s^o \in \chi} \|h(s^o)\|$. To facilitate the analysis, let $\{\varpi_j \in \mathbb{R}_{>0} \mid j = 1, \cdots, 7\}$ be constants such that $\varpi_1 + \varpi_2 + \varpi_3 = 1$, and $\varpi_4 + \varpi_5 + \varpi_6 + \varpi_7 = 1$. Let $\underline{c} \in \mathbb{R}_{>0}$ be a constant defined as

$$\underline{c} := \frac{\beta}{2\overline{\Gamma} k_{c2}} + \frac{\underline{c}_3}{2}, \tag{41}$$

k_5 be a positive constant defined as $k_5 := \bar{W} K_{c1} \overline{\|\nabla_s \sigma\|} L_y$ and $\iota \in \mathbb{R}$ be a positive constant defined as

$$\iota \triangleq \frac{(k_{c1} + k_{c2})^2 \overline{\|\Delta\|}^2}{4k_{c2}\underline{c}\varpi_3} + \frac{1}{4}\overline{\|G_\epsilon\|} + \frac{1}{4(k_{a1} + k_{a2})\varpi_6}\left(\frac{1}{2}\overline{W}\overline{\|G_\sigma\|} + \frac{1}{2}\overline{\|\nabla_s \epsilon G^T \nabla_s \sigma^T\|}\right) + \frac{1}{4(k_{a1} + k_{a2})\varpi_6}\left(k_{a2}\overline{W} + \frac{1}{4}(k_{c1} + k_{c2})\overline{W}^2 \overline{\|G_\sigma\|}\right)^2. \tag{42}$$

To facilitate the stability analysis, let $V_L : \mathbb{R}^{n+2L+p} \times \mathbb{R}_{\geq 0} \to \mathbb{R}_{\geq 0}$ be a continuously differentiable candidate Lyapunov function defined as

$$V_L(Z, t) := V^*(s) + \frac{1}{2}\tilde{W}_c^T \Gamma^{-1}(t) \tilde{W}_c + \frac{1}{2}\tilde{W}_a^T \tilde{W}_a + V_1(\tilde{\theta}), \tag{43}$$

where V^* is the optimal value function, V_1 was introduced in section 4 and $Z \triangleq \left[s;\ \tilde{W}_c;\ \tilde{W}_a;\ \tilde{\theta}\right]$. The update law in (29) ensures that the adaptation gain matrix is bounded such that

$$\underline{\Gamma} \leq \|\Gamma(t)\| \leq \overline{\Gamma}, \forall t \in \mathbb{R}_{\geq T}. \tag{44}$$

Using the fact that V^* and V_1 are positive definite, Lemma 4.3 from Khalil (2002) yields

$$\underline{v_l}\left(\|Z\|\right) \leq V_L\left(Z,t\right) \leq \overline{v_l}\left(\|Z\|\right), \tag{45}$$

for all $t \in \mathbb{R}_{\geq T}$ and for all $Z \in \mathbb{R}^{n+2L+p}$, where $\underline{v_l}, \overline{v_l} : \mathbb{R}_{\geq 0} \to \mathbb{R}_{\geq 0}$ are class $\mathcal{K}$ functions. Let $v_l : \mathbb{R}_{\geq 0} \to \mathbb{R}_{\geq 0}$ be a function defined as $v_l\left(\|Z\|\right) := \frac{\lambda_{\min}\{Q\}\|s\|^2}{2} + \frac{k_{c2}\underline{c}\varpi_1}{2}\left\|\tilde{W}_c\right\|^2 + \frac{(k_{a1}+k_{a2})\varpi_4}{2}\left\|\tilde{W}_a\right\|^2 + \frac{\|\tilde{\theta}\|^2}{2}$.

The sufficient conditions for ultimate boundedness of Z are derived based on the subsequent stability analysis as

$$\left(k_{c2}\underline{c}\varpi_2 - \frac{k_5 r\epsilon}{2}\right)(k_{a1}+k_{a2})\varpi_5 \geq \left(k_{a1} + \frac{1}{4}\left(k_{c1}+k_{c2}\right)\overline{W}\overline{\|G_\sigma\|}\right), \tag{46}$$

$$\left(k_{a1}+k_{a2}\right)\varpi_7 \geq \frac{1}{4}\left(k_{c1}+k_{c2}\right)\overline{W}\overline{\|G_\sigma\|}, \tag{47}$$

$$\lambda_{\min}\{Y_f(T)\} \geq \frac{k_5 r}{2\epsilon} + 1, \tag{48}$$

$$v_l^{-1}(\iota) < \overline{v_l}^{-1}(\underline{v_l}(r)). \tag{49}$$

The bound on the function F and the NN function approximation errors depend on the underlying compact set; hence, ι is a function of r. Even though, in general, ι increases with increasing r, the sufficient condition in (49) can be satisfied provided the points for BE extrapolation are selected such that the constant $\underline{c}$, introduced in (41) is large enough and that the basis for value function approximation are selected such that $\overline{\|\epsilon\|}$ and $\overline{\|\nabla\epsilon\|}$ are small enough.

The differential equation (7), under the controller in (32), along with (12), (29), and (31), constitute the closed-loop system $\dot{Z} = h(Z,t)$ to be analyzed. Let $\dot{V}_L$ denote the orbital derivative of (43) along the trajectories of the closed-loop system, i.e., $\dot{V}_L := \nabla_t V_L + \nabla_Z V_L(Z,t)h(Z,t)$. Then,

$$\dot{V}_L = \nabla_s V^* F + \nabla_s V^* G\hat{u} + \tilde{W}_c^T \Gamma^{-1}\dot{\tilde{W}}_c + \frac{1}{2}\tilde{W}_c^T \dot{\Gamma}^{-1}\tilde{W}_c + \tilde{W}_a^T \dot{\tilde{W}}_a + \dot{V}_1. \tag{50}$$

Substituting (29) - (31) in (50) yields

$$\begin{aligned}\dot{V}_L \leq \nabla_s V^*\left(F + Gu^*\right) - \nabla_s V^* G u^* + \nabla_s V^* G\hat{u} - \tilde{W}_c^T\Gamma^{-1}\left(-k_{c1}\Gamma\frac{\omega}{\rho}\hat{\delta} - \frac{1}{N}\Gamma\sum_{k=1}^{N}\frac{k_{c2}\omega_i}{\rho_k}\hat{\delta}_k\right)\\ - \frac{1}{2}\tilde{W}_c^T\Gamma^{-1}\left(\beta\Gamma - k_{c1}(\Gamma\frac{\omega\omega^T}{\rho^2}\Gamma) - \frac{k_{c2}}{N}\Gamma\sum_{k=1}^{N}\frac{\omega_k\omega_k^T}{\rho_k^2}\Gamma\right)\Gamma^{-1}\tilde{W}_c\\ - \tilde{W}_a^T\left(-k_{a1}(\hat{W}_a - \hat{W}_c) - k_{a2}\hat{W}_a + \left((\frac{k_{c1}\omega}{4\rho}\hat{W}_a^T G_\sigma + \sum_{k=1}^{N}\frac{k_{c2}\omega_k}{4N\rho_k}\hat{W}_a^T G_{\sigma k})^T\hat{W}_c\right)\right) + \dot{V}_1.\end{aligned} \tag{51}$$

Using Young's inequality, Cauchy-Schwarz inequality, and completion of squares, (51) can be bounded as

$$\dot{V}_L \leq -s^T Q s - k_{c2}\underline{c}\left(\varpi_1 + \varpi_2 + \varpi_3\right)\left\|\tilde{W}_c\right\|^2 - \left(k_{a1}+k_{a2}\right)\left(\varpi_4 + \varpi_5 + \varpi_6 + \varpi_7\right)\left\|\tilde{W}_a\right\|^2$$

$$+\left(\frac{1}{2}\overline{W}\overline{\|G_\sigma\|}+\frac{1}{2}\left\|\nabla_s\epsilon G^T\nabla_s\sigma^T\right\|+k_{a2}\overline{W}+\frac{1}{4}\left(k_{c1}+k_{c2}\right)\overline{W}^2\overline{\|G_\sigma\|}\right)\left\|\tilde{W}_a\right\|$$

$$+\left\|\tilde{W}_c\right\|\left(\left(k_{c1}+k_{c2}\right)\left\|\hat{\delta}\right\|\right)+\left(k_{a1}+\frac{1}{4}\left(k_{c1}+k_{c2}\right)\overline{W}\overline{\|G_\sigma\|}\right)\left\|\tilde{W}_a\right\|\left\|\tilde{W}_c\right\|+\frac{1}{4}\overline{\|G_\epsilon\|}$$

$$+\frac{1}{4}\left(k_{c1}+k_{c2}\right)\overline{W}\overline{\|G_\sigma\|}\left\|\tilde{W}_a\right\|^2-\lambda_{\min}\{Y_f\}\|\tilde{\theta}\|^2+\left(k_5 r\right)\left(\frac{\|\tilde{\theta}\|^2}{2\epsilon}+\frac{\epsilon\|\tilde{W}_c\|^2}{2}\right). \quad (52)$$

Provided the gains are selected based on the sufficient conditions in (46), (47), (48) and (49), the orbital derivative can be upper-bounded as

$$\dot{V}_L\leq-v_l\left(\|Z\|\right),\quad\forall\,\|Z\|\geq v_l^{-1}\left(\iota\right), \quad (53)$$

for all $t\geq T$ and $\forall Z\in B_r$. Using (45), (49), and (53), Theorem 4.18 in Khalil (2002) can then be invoked to conclude that Z is locally uniformly ultimately bounded in the sense that all trajectories starting from initial conditions bounded by $\|Z(T)\|\leq\overline{v_l}^{-1}(\underline{v_l}(r))$, satisfy $\limsup_{t\to\infty}\|Z\left(t\right)\|\leq\underline{v_l}^{-1}\left(\overline{v_l}\left(v_l^{-1}\left(\iota\right)\right)\right).$ Furthermore, the concatenated state trajectories are bounded such that $\|Z\left(t\right)\|\in B_r$ for all $t\in\mathbb{R}_{\geq T}$. Since the estimates $\hat{W}_a$ approximate the ideal weights W, the policy $\hat{u}$ approximates the optimal policy u^*.
